# Association between HOMA-IR and metabolic dysfunction-associated steatohepatitis in U.S. adults with MASLD

**DOI:** 10.1016/j.metop.2025.100402

**Published:** 2025-09-29

**Authors:** Xiao-Xuan Tang, Rui Wu, Jun-Hui Chen, Feng-Lan Wang, Sai-Li Zhao, Jie Lu, Jian Qin, Duan-Ming Zhuang, Bin Zhang

**Affiliations:** a Department of Gastroenterology, Nanjing Drum Tower Hospital, Affiliated Hospital of Medical School, Nanjing University, Nanjing, China; b Department of Gastroenterology, Fengyang County People's Hospital, Chuzhou, China; c Department of Gastroenterology, Bingtuan Sishi Hospital, Ili, China; d Department of Gastroenterology, Gaochun People's Hospital, Nanjing, China

## Abstract

**Background:**

MASH is a critical point in metabolic dysfunction-associated steatotic liver disease (MASLD). Understanding its association with the Homeostatic Model Assessment of Insulin Resistance (HOMA-IR) is essential, as HOMA-IR is a marker for insulin resistance.

**Methods:**

This study analyzed 700 adults from the NHANES 2017–2020, using the FAST score (with thresholds of ≥0.35 and ≥ 0.67) to identify individuals at high MASH risk. Logistic regression assessed HOMA-IR's association with MASH risk, while linear regression evaluated its link to liver stiffness measurement (LSM) and controlled attenuation parameter (CAP). Nonlinear associations were explored using restricted cubic splines (RCS), and BMI's mediation effects were examined through causal mediation analysis.

**Results:**

MASH risk was significantly higher in the highest HOMA-IR quartile compared to the lowest (OR = 5.942, 95 %CI = 2.117–16.679, P = 0.001). RCS revealed nonlinear associations between HOMA-IR and both MASH risk (P = 0.007) and liver metrics (LSM: P = 0.045; CAP: P < 0.001). HOMA-IR correlated with increased hepatic steatosis and fibrosis severity. BMI mediated 34.26 % and 19.62 % of the associations for LSM and CAP, respectively.

**Conclusion:**

Monitoring HOMA-IR is vital for early MASH risk detection and intervention. Targeting insulin resistance and BMI may reduce MASH risk and severity, highlighting the need for integrated therapeutic strategies.

## List of abbreviations

Adipo-IR: Adipose tissue insulin resistance
ALT: Alanine aminotransferase
AST: Aspartate aminotransferase
BMI: Body mass index
CAP: Controlled attenuation parameter
DNL: De novo lipogenesis
FAST: FibroScan Assessment of Steatohepatitis
FFA: Free fatty acids
FPG: Fasting blood glucose
FIB-4: Fibrosis-4
GDM: Gestational diabetes mellitus
GLM: Generalized linear model
HbA1c: Hemoglobin A1c
HOMA-IR: Homeostatic Model Assessment of Insulin Resistance
IR: Insulin resistance
LSM: Median liver stiffness
MASH: Metabolic dysfunction-associated steatohepatitis
MASLD: Metabolic dysfunction-associated steatotic liver disease
MET: Metabolic Equivalent
NAS: NAFLD Activity Score
NAFLD: Non-alcoholic fatty liver disease
NASH: Non-alcoholic steatohepatitis
NHANES: National Health and Nutrition Examination Survey
OGTT: Oral glucose tolerance tests
PSM: Propensity score matching
QUICKI: Quantitative Insulin Sensitivity Check Index
RCS: Restricted cubic spline
RIP: Ratio of family income to poverty
ROS: Reactive oxygen species
VCTE: Vibration-controlled transient elastography

## 1 Introduction

Non-alcoholic fatty liver disease (NAFLD) is a prevalent chronic liver disease globally, evolving from simple steatosis to non-alcoholic steatohepatitis (NASH), with complications including cirrhosis and hepatocellular carcinoma [1]. A newly proposed term, metabolic dysfunction-associated steatotic liver disease (MASLD), aims to replace NAFLD due to its inclusive criteria that better reflect the disease's pathophysiology and its metabolic implications [2]. The definition of MASLD is more inclusive and performs higher sensitivity in lean centrally obese non-diabetic patients with NAFLD [3]. Similarly, metabolic dysfunction-associated steatohepatitis (MASH) is suggested to replace NASH. MASH, an inflammatory subtype of MASLD, is associated with increased risks of cirrhosis and the need for liver transplantation, primarily driven by global rises in obesity and metabolic syndrome [[4], [5], [6]]. Insulin resistance (IR) plays a crucial role in the development of MASH, contributing to hepatic steatosis, inflammation, and fibrosis [7]. During insulin resistance, while insulin-mediated suppression of gluconeogenesis in the liver is impaired, an increase in de novo lipogenesis (DNL) persists. This imbalance contributes to increased hepatic fat deposition, which can impair mitochondrial function in hepatocytes, trigger the release of reactive oxygen species (ROS), and exacerbate hepatitis [8,9]. The Homeostatic Model Assessment of Insulin Resistance (HOMA-IR), a widely used indicator of IR, correlates with obesity, creating a cycle that exacerbates liver damage [6,[10], [11], [12], [13]]. Taken together, the existing evidence suggests that IR predisposes individuals to obesity, which often leads to an adverse impact on liver steatosis and fibrosis. It appears that these IR-related conditions might in part be responsible for the undesirable effect of IR on liver steatosis and fibrosis. The pivotal role of IR in metabolic dysregulation is further highlighted by its connections to various disorders. Genetic variants like CDKAL1 increase gestational diabetes mellitus (GDM) risk, a condition pathophysiologically linked to MASLD [14]. Similarly, elevated Galectin-3 in GDM underscores inflammation's role [15]. IR-related systemic effects are also reflected in hematological parameters [16] and thyroid-related fertility impairment, reinforcing IR as a central metabolic driver [17,18].

While HOMA-IR was excluded from MASLD diagnostic criteria due to practical considerations [19], it remains a robust research tool for investigating insulin resistance pathophysiology, highlighting a gap in assessing MASH risk in MASLD patients. Previous studies have confirmed that HOMA-IR is closely linked to the development of NAFLD in non-diabetic populations, including its role in predicting liver fibrosis progression [20,21], diagnosing NAFLD [22], and offering a comparative advantage over other metabolic indicators [23]. For instance, a comparative study demonstrated that HOMA-IR had superior specificity and accuracy to the Quantitative Insulin Sensitivity Check Index (QUICKI) in diagnosing MASLD [24], and recent large-scale analyses further corroborate HOMA-IR's advantage as a more robust predictor for NAFLD [25]. However, there is a lack of in-depth research on the specific association between HOMA-IR and MASH in individuals with MASLD, particularly its gradient effect on the severity of hepatic steatosis and fibrosis, as well as the mediating role of BMI. Our study focuses on the relationship between IR, obesity, and liver damage, using data from the MASLD population in the National Health and Nutrition Examination Survey (NHANES). By understanding this relationship and its impact on liver steatosis and fibrosis, we aim to improve the prevention, treatment, and prevalence assessment of MASH.

## 2 Materials and methods

### 2.1 Data source and study population

**Fig. 1:**
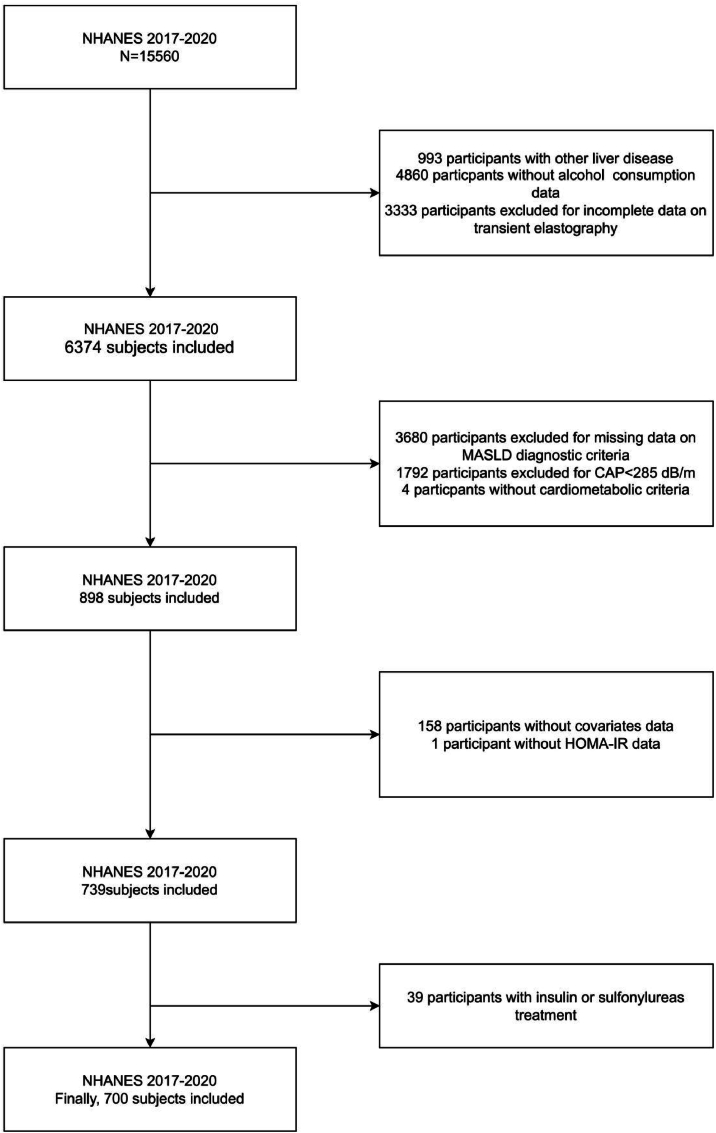
Flowchart of participant selection.

The NHANES data, accessible at www.cdc.gov/nchs/nhanes/, is available gratis to the global research community [26]. The study initially considered 15,560 participants, but analysis was confined to 700 participants due to specific exclusion criteria: presence of other liver diseases or incomplete data related to alcohol consumption and transient elastography (9186 excluded), lack of data aligning with MASLD diagnostic criteria (5476 excluded), insufficient data on HOMA-IR or other covariates (159 excluded), and participants undergoing treatment with insulin or Sulfonylureas (39 excluded). This research adhered to ethical standards, receiving approval from the NCHS Research Ethics Review Board and participant consent was secured through signed consent forms. The process of participant selection is illustrated in Fig. 1.

### 2.2 Covariates

This study included several covariates: age, gender, race, educational level, ratio of family income to poverty (RIP), body mass index (BMI), abdominal obesity, smoking status, alcohol consumption, physical activity, and health conditions such as hypertension and diabetes. Racial categories were defined as Mexican American, Other Hispanic, Non-Hispanic Black, Non-Hispanic White, and Other races/ethnicities. Educational attainment was categorized into less than high school, high school graduate, and more than high school. Economic status was measured using the RIP [27]. BMI was calculated as weight in kilograms divided by height in meters squared, with abdominal obesity defined as a waist circumference of ≥88 cm for women and ≥102 cm for men [28]. Smoking status was assessed by past and current smoking habits while daily alcohol intake was evaluated through 24-h dietary recall interviews. Hypertension was identified through self-report, medication use, or blood pressure readings exceeding 140/90 mmHg [29]. Diabetes was diagnosed by self-report, use of medication to control blood glucose, fasting blood glucose(FPG) levels of 7 mmol/L or higher, or hemoglobin A1c(HbA1c) values of 6.5 % or higher [30]. Prediabetes was determined by FPG level of 5.6–6.9 mmol/L, HbA1c values of 5.7–6.4 % for no diabetes people [31]. Physical activity was quantified using Metabolic Equivalent (Task MET) scores, frequency, and duration of activities, based on the method described by Xiong Lei et al. [32]. The formula used for calculation was: PA (MET-hours/week) = MET value × weekly frequency × duration per session.

### 2.3 Calculation of HOMA-IR

The Homeostatic Model Assessment of Insulin Resistance (HOMA-IR) is a widely recognized mathematical model for evaluating insulin resistance, calculated from fasting blood glucose and insulin levels using the formula: HOMA-IR = (fasting glucose [mmol/L] × fasting insulin [μU/mL])/22.5 [25]. To avoid skewed results from medication effects, our study excluded participants on insulin or sulfonylurea treatments. We divided study participants into quartiles based on their HOMA-IR values, with Quartile 1 (Q1) ranging from 0.50 to 2.85, Quartile 2 (Q2) from 2.86 to 4.63, Quartile 3 (Q3) from 4.64 to 7.40, and Quartile 4 (Q4) from 7.41 to 65.20.

### 2.4 Diagnosis of MASLD and MASH

The diagnostic criteria for Metabolic Dysfunction-Associated Steatotic Liver Disease (MASLD) are grounded in an international consensus, requiring simultaneous identification of hepatic steatosis (CAP value ≥ 285 dB/m), exclusion of other liver diseases including excessive alcohol intake (more than 20g per day for women and 30g per day for men), and meeting at least one cardiometabolic criterion. These criteria include: a BMI ≥25.0 kg/m^2^ or waist circumference of ≥94 cm for men or ≥80 cm for women; fasting plasma glucose ≥5.6 mmol/L or equivalent indicators of glucose intolerance; blood pressure ≥130/85 mmHg or on antihypertensive medication; plasma triglycerides ≥1.7 mmol/L or using lipid-lowering drugs; and low plasma high-density lipoprotein cholesterol (HDL-C: <40 mg/dL for men, <50 mg/dL for women) or on specific lipid-lowering treatment [6]. For assessing the risk of MASH, our study utilized the Fatty Liver Fibroscan Assessment (FAST) score combining LSM, CAP, and AST levels. This score, calculated based on methodologies by Philip N Newsome et al., employs thresholds of 0.35 (90 % sensitivity) and 0.67 (90 % specificity) to gauge MASH prevalence effectively [33]. Federico's meta-analysis demonstrated that the FAST score is effective in the non-invasive detection of fibrotic NASH, exhibiting high sensitivity and specificity rates of 89 % (95 % CI: 82 %–93 %) and 89 % (95 % CI: 83 %–94 %) at the exclusion threshold (≤0.35) and diagnostic threshold (≥0.67), respectively. It also reported a very high negative predictive value of 92 % at the exclusion threshold. Subgroup analyses and impact bias assessments did not alter these findings [34].NHANES' 2017–2020 cycle employed vibration-controlled transient elastography (VCTE) to measure liver fibrosis and steatosis, with a CAP value of ≥285 dB/m confirming steatosis [35]. LSM data was also collected to evaluate liver fibrosis severity. This method has been validated in numerous studies for its accuracy in detecting liver conditions non-invasively.

### 2.5 Statistical analysis

According to the NHANES guidelines, in the process of analyzing NHANES data, we considered the complex sampling designs and sampling weights [36]. In this study, categorical variables were analyzed using frequency and weighted proportions, while continuous variables were described using means ± standard deviation or median (interquartile range). We employed weighted multivariate logistic regression to explore the relationship between HOMA-IR and MASH, adjusting for covariates such as age, gender, race, education, income, abdominal obesity, smoking, alcohol consumption, hypertension, diabetes, and physical activity. Additionally, weighted multivariate linear regression was used to assess the relationship between HOMA-IR and LSM, and CAP. To ensure the robustness of our findings, we conducted sensitivity analyses using propensity score matching (PSM), which was based on age, gender, and BMI, using a 1:2 matching ratio and a caliper width of 0.2 standard deviations. The associations between HOMA-IR and MASH, LSM, and CAP were re-evaluated using weighted logistic and linear regressions post-PSM. Nonlinear relationships were modeled with restricted cubic splines (3 knots at the 5th, 50th, and 95th percentiles). Furthermore, mediation analysis was performed to determine the role of BMI in the association between HOMA-IR and LSM/CAP. This involved constructing a mediator model to evaluate the relationship between HOMA-IR (exposure) and BMI (mediator) and an outcome model assessing the combined effects of the exposure and mediator on LSM/CAP (outcomes). The mediation effect's significance was tested using Bootstrap sampling with 500 iterations [37]. Data analysis was performed using R software (version 4.3.2). A two-sided P-value <0.05 was considered statistically significant.

## 3 Results

### 3.1 Baseline characteristics

Our study included 700 participants, divided into 345 men and 355 women, whose baseline characteristics are detailed in Table 1. Participants identified as high risk for MASH were notably younger, predominantly male, and exhibited significantly higher BMI and HOMA-IR values (BMI: 35.438 vs 32.682; HOMA-IR: 7.427 vs 3.997), compared to their counterparts. These high risk individuals also showed elevated CAP and LSM values (CAP: 365.105 vs 321.000; LSM: 7.954 vs 5.400). Following 1:2 PSM, 277 individuals were successfully matched between the non-MASH (n = 181) and high risk MASH (n = 96) groups. Post-PSM analysis indicated no significant disparities in age, gender, and BMI between groups, validating the effectiveness of the matching process. However, the high risk MASH group continued to show significantly greater HOMA-IR, CAP, and LSM levels after matching, indicating intrinsic differences between groups.

**Table 1:** General characteristics of participants with high risk MASH (FAST Score ≥0.35)^a^ before and after PSM in the NHANES 2017–2020.

Characters	Before PSM (n = 700)			After PSM (n = 277)		
	FAST score <0.35 (n = 603)	FAST score ≥0.35 (n = 97)	P value	FAST score <0.35 (n = 181)	FAST score ≥0.35 (n = 96)	P value
**Age(years)**	54.000(39.000–65.000)	46.000(33.000–57.000)	**0.0028**	47.000(33.000–61.000)	46.000(33.752–56.643)	0.3549
**Gender**			**0.0083**			0.2373
**Man**	284(46.20 %)	61(66.66 %)		108(57.10 %)	60(66.32 %)	
**Woman**	319(53.80 %)	36(33.34 %)		73(42.90 %)	36(33.68 %)	
**Race**			0.3826			0.4926
**Mexican American**	98(10.17 %)	20(13.87 %)		31(10.79 %)	20(14.01 %)	
**Other Hispanic**	53(5.06 %)	10(9.23 %)		14(4.39 %)	10(9.32 %)	
**Non-Hispanic White**	248(69.00 %)	34(59.96 %)		76(68.47 %)	33(59.55 %)	
**Non-Hispanic Black**	126(8.29 %)	17(8.10 %)		44(9.28 %)	17(8.19 %)	
**Other Race**	78(7.48 %)	16(8.84 %)		16(7.07 %)	16(8.93 %)	
**Education**			0.7639			0.6398
**<high school**	94(9.17 %)	15(7.91 %)		35(11.37 %)	15(7.99 %)	
**High school**	156(28.15 %)	18(25.13 %)		44(27.39 %)	17(1 24.36 %)	
**>high school**	353(62.68 %)	64(66.96 %)		102(61.24 %)	64 67.65 %)	
**Ratio of family income to poverty**	3.059(1.817–5.000)	2.552(1.400–4.341)	0.2429	2.988(1.887–4.126)	2.554(1.423–4.355)	0.5322
**BMI(kg/m2)**	32.682(28.900–37.300)	35.438(32.100–42.333)	**0.0096**	35.500(31.131–39.289)	35.422(32.100–42.268)	0.6016
**Abdominal obesity**			0.0881			0.3800
**No**	93(14.69 %)	11(6.98 %)		23(10.94 %)	11(7.05 %)	
**Yes**	510(85.31 %)	86(93.02 %)		158(89.06 %)	85(92.95 %)	
**Transient Elastography**						
**Median liver stiffness(kPa)**	5.400(4.400–6.531)	7.954(6.200–10.700)	**<0.0001**	5.314 (4.500–6.192)	7.900 (6.200–10.389)	**<0.0001**
**Controlled attenuation parameter(dB/m)**	321.000(300.958–346.000)	365.105(328.988–392.000)	**<0.0001**	320.000(302.000–346.000)	363.000(328.794–390.939)	**<0.0001**
**Smoker**			0.5229			0.5023
**No**	341(61.99 %)	61(58.88 %)		109(64.14 %)	60(58.46 %)	
**Yes**	262(38.01 %)	36(41.12 %)		72(35.86 %)	36(41.54 %)	
**Alcohol(g/d)**	2.656 ± 6.106	2.610 ± 6.468	0.9629	2.573 ± 6.195	2.517 ± 6.434	0.9576
**Hypertension**			0.1053			0.1331
**No**	262(50.75 %)	36(36.79 %)		80(50.28 %)	35(36.14 %)	
**Yes**	341(49.25 %)	61(63.21 %)		101(49.72 %)	61(63.86 %)	
**Diabetes**			0.0948			**0.0331**
**No diabetes**	105(21.80 %)	9(10.92 %)		35(20.37 %)	9(11.03 %)	
**Prediabetes**	297(51.01 %)	41(48.18 %)		89(58.83 %)	41(48.67 %)	
**Diabetes**	201(27.19 %)	47(40.90 %)		57(20.79 %)	46(40.29 %)	
**Physical activity(MET-minute/week)**	720.000(0.000–4320.000)	1121.115(0.000–5354.882)	0.5071	526.487(0.000–6000.000)	1141.743(0.000–5437.096)	0.6465
**HOMA-IR**	3.997 (2.502–6.357)	7.427 (4.862–12.732)	**<0.0001**	4.176 (2.502–6.589)	7.196 (4.861–12.069)	**0.0001**
**HOMA-IR Quartiles**			**<0.0001**			**0.0007**
**Quartiles 1(0.50**–**2.85)**	166(31.81 %)	11(7.26 %)		41(31.53 %)	11(7.33 %)	
**Quartiles 2(2.86**–**4.63)**	166(27.36 %)	9(13.80 %)		45(26.18 %)	9(13.94 %)	
**Quartiles 3(4.64**–**7.40)**	149(21.92 %)	26(28.52 %)		54(23.06 %)	26(28.82 %)	
**Quartiles 4(7.41**–**65.20)**	122(18.91 %)	51(50.41 %)		41(19.23 %)	50(49.91 %)	

P-values are weighted.Bold indicates P value < 0.05. PSM: propensity score matching; BMI: body mass index; HOMA-IR: homeostatic model assessment for insulin resistance.a.

a FAST score is intended to identify individuals with MASH. A cutoff of 0.35 yields a sensitivity of 0.90.

### 3.2 Associations between HOMA-IR level and the prevalence of MASH

**Fig. 2:**
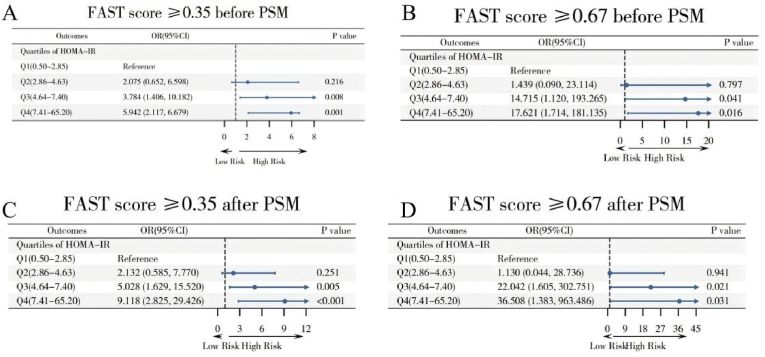
Association between HOMA-IR and the prevalence of MASH before (Panel A and B: using FAST score cutoffs of ≥0.35 and ≥ 0.67) and after (Panel C and D: using FAST score cutoffs of ≥0.35 and ≥ 0.67) PSM. The weighted logistic regression analysis adjusted for gender; age; race; education; ratio of family income to poverty; BMI; abdominal obesity; smoker; alcohol; hypertension; diabetes; physical activity.

**Fig. 3:**
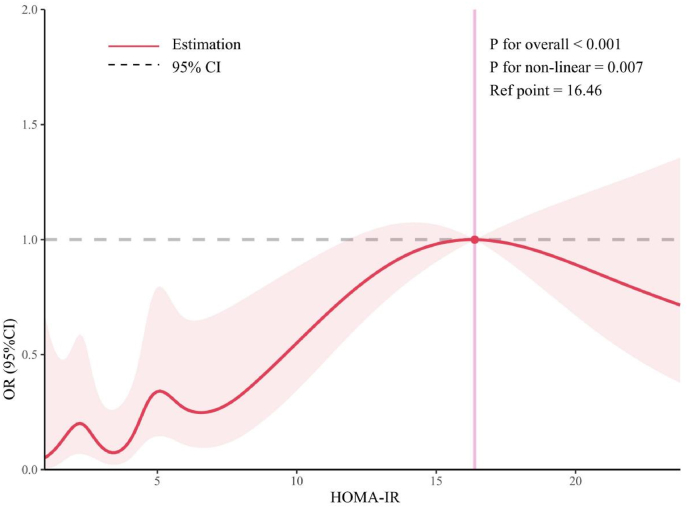
Examination of the nonlinear association between HOMA-IR and the prevalence of MASH using a FAST score of ≥0.35. The RCS model adjusted for gender; age; race; education; ratio of family income to poverty; BMI; smoker; alcohol; hypertension; diabetes and physical activity.

Fig. 2 illustrates that after adjusting for variables such as gender, age, race, education, income, BMI, abdominal obesity, smoking, alcohol consumption, hypertension, diabetes, and physical activity, the adjusted multivariate logistic regression shows a significant association between HOMA-IR and the overall prevalence of high-risk MASH, as determined using FAST scores of ≥0.35 and ≥ 0.67. For Q3 and Q4, the odds ratios (ORs) are significantly higher compared to Q1. Specifically, for a FAST score of ≥0.35, the OR is 3.784 (95 %CI = 1.406–10.182) for Q3 and 14.715 (95 %CI = 1.120–193.265) for Q4. For a 10.13039/100020758FAST score of ≥0.67, the OR increases to 5.942 (95 %CI = 2.117–16.679) for Q3 and 17.621 (95 %CI = 1.714–181.135) for Q4. PSM analysis further supports these findings, demonstrating even higher ORs in Q3 and Q4 relative to Q1. For a FAST score of ≥0.35, the OR post-PSM is 5.028 (95 %CI = 1.629–15.520) for Q3 and 22.042 (95 %CI = 1.605–302.751) for Q4; for a FAST score of ≥0.67, the OR post-PSM is 9.118 (95 %CI = 2.825–29.426) for Q3 and 36.508 (95 %CI = 1.383–963.486) for Q4. The unadjusted analysis yielded consistent findings, with significantly elevated odds of high-risk MASH in the highest HOMA-IR quartiles (Q3 and Q4) compared to Q1 (Supplementary Fig. 1). Additionally, RCS analysis, depicted in Fig. 3, identifies a non-linear association between HOMA-IR and high-risk MASH (P for non-linear = 0.007), with a notable threshold effect at a HOMA-IR value of 16.46. Below this threshold, the OR is 1.230 (95 %CI = 1.090–1.388), indicating a significant association. However, beyond this threshold, the association diminishes (HOMA-IR > 16.46: OR = 0.943, 95 %CI = 0.795–1.118), as shown in Table 2, signifying that higher HOMA-IR values do not correlate with an increased prevalence of high-risk MASH.

**Table 2:** Threshold effect analysis of HOMA-IR on the prevalence of MASH using the two-piecewise linear regression model.

MASH using a FAST score of ≥0.35 (n = 97)	
Parameters	Adjusted OR(95 %CI),P-value
**All participants**	
Fitting by the standard linear model	**1.082(1.018,1.149)0.011**
**Fitting by the two-piecewise linear model**	
Inflection point	16.46
HOMA-IR≤16.46	**1.230(1.090,1.388)0.001**
HOMA-IR>16.46	0.943(0.795,1.118)0.498

Adjusted for:gender; age; race; education; ratio of family income to poverty; BMI; abdominal obesity; smoker; alcohol; hypertension; diabetes; physical activity.

### 3.3 Associations between HOMA-IR level and LSM

**Fig. 4:**
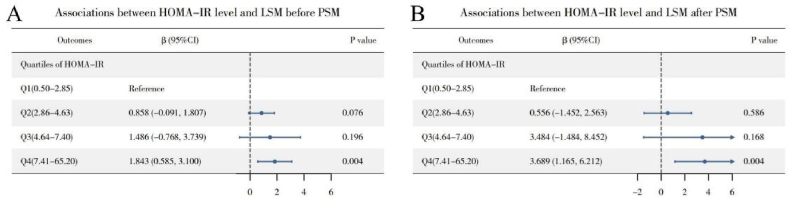
Association between HOMA-IR and LSM before (Panel A) and after (Panel B) PSM. The weighted logistic regression analysis adjusted for gender; age; race; education; ratio of family income to poverty; abdominal obesity; smoker; alcohol; hypertension; diabetes; physical activity.

**Fig. 5:**
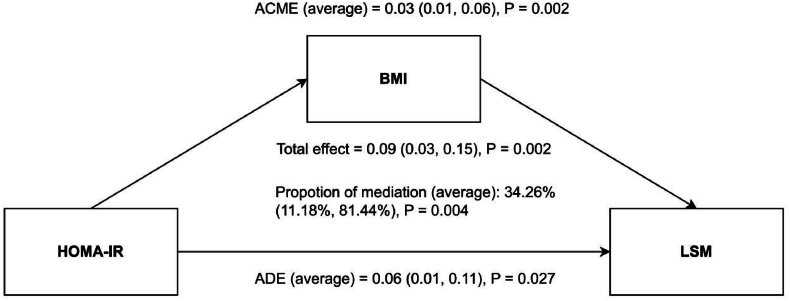
Path diagram of the mediation of BMI on the association between HOMA-IR on LSM. ACME, average causal mediation effects; ADE, average direct effects.

**Fig. 6:**
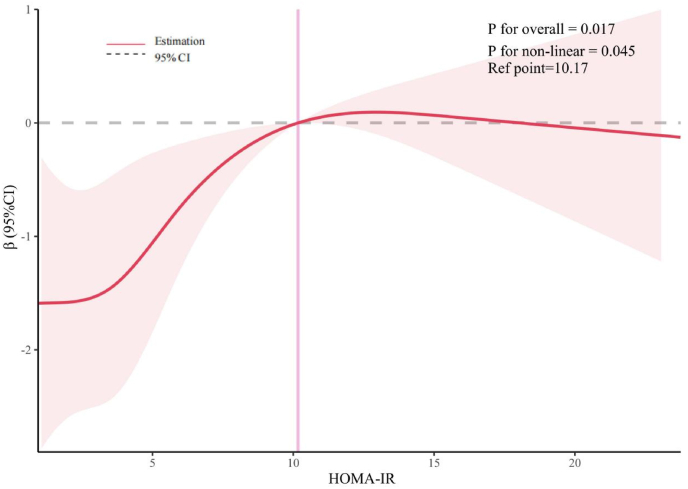
Examination of the nonlinear association between HOMA-IR and LSM. The RCS model adjusted for gender; age; race; education; ratio of family income to poverty; smoker; alcohol; hypertension; diabetes and physical activity.

Fig. 4 presents results from a multivariate linear regression analysis, adjusted for gender, age, race, education, income, abdominal obesity, smoking, alcohol consumption, hypertension, diabetes, and physical activity. It shows that LSM values are significantly higher in the highest insulin resistance quartile, Q4, compared to Q1, with a coefficient (β) of 1.843 (95 %CI = 0.585–3.100). Post-PSM, the difference in LSM between Q4 and Q1 further increases, denoted by a β of 3.689 (95 %CI = 1.165–6.212). To elucidate the pathway underlying the association between HOMA-IR and LSM, a mediation analysis was performed (Fig. 5). The total effect of HOMA-IR on LSM was significant (β = 0.09, 95 % CI: 0.03 to 0.15, P = 0.002). This effect was decomposed into a significant average direct effect (ADE) (β = 0.06, 95 % CI: 0.01 to 0.11, P = 0.027), representing the effect not mediated by BMI, and a significant average causal mediation effect (ACME) (β = 0.03, 95 % CI: 0.01 to 0.06, P = 0.002), representing the effect mediated through BMI. The proportion of mediation was 34.26 %. The significance of the ADE indicates that BMI partially mediates the association between HOMA-IR and LSM. The results of the mediation analysis are shown in Fig. 5. RCS analysis,as depicted in Fig. 6, indicated a non-linear relationship between HOMA-IR and LSM (P for non-linear = 0.045), identifying a threshold effect at a HOMA-IR value of 10.17. Below this threshold, the association remains positive (β = 0.276, 95 %CI = 0.111–0.441), whereas above it, the association is not significant (β = −0.010, 95 %CI = −0.142-0.123), as evidenced in Table 3. These findings highlight that high HOMA-IR values do not correlate with increased LSM once a certain threshold is surpassed.

**Table 3:** Threshold effect analysis of HOMA-IR on the Median liver stiffness using the two-piecewise linear regression model.

LSM	
Parameters	Adjusted β(95 %CI),P-value
**All participants**	
Fitting by the standard linear model	**0.088(0.003,0.172)0.042**
**Fitting by the two-piecewise linear model**	
Inflection point	10.17
HOMA-IR≤10.17	**0.276(0.111,0.441)0.001**
HOMA-IR>10.17	−0.010(-0.142,0.123)0.886

Adjusted for:gender; age; race; education; ratio of family income to poverty; smoker; alcohol; hypertension; diabetes; physical activity.

### 3.4 Associations between HOMA-IR level and CAP

**Fig. 7:**
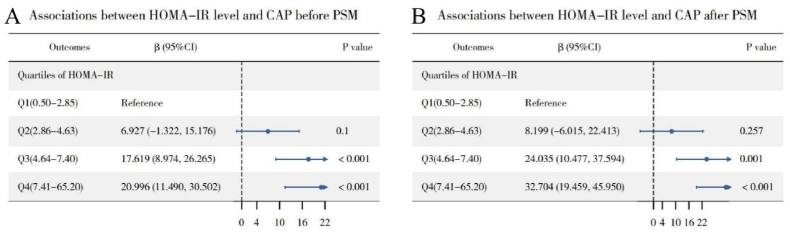
Association between HOMA-IR and CAP before (Panel A) and after (Panel B) PSM. The weighted logistic regression analysis adjusted for gender; age; race; education; ratio of family income to poverty; abdominal obesity; smoker; alcohol; hypertension; diabetes; physical activity.

**Fig. 8:**
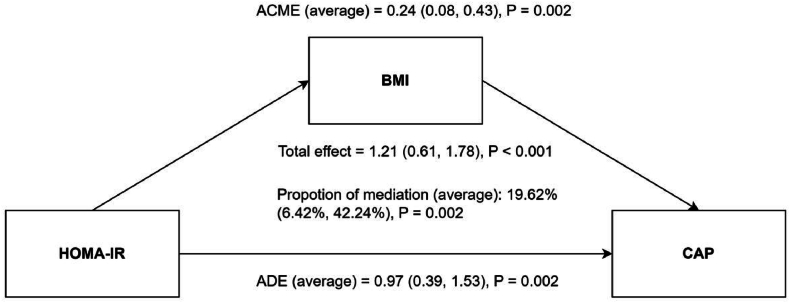
Path diagram of the mediation of BMI on the association between HOMA-IR on CAP. ACME, average causal mediation effects; ADE, average direct effects.

**Fig. 9:**
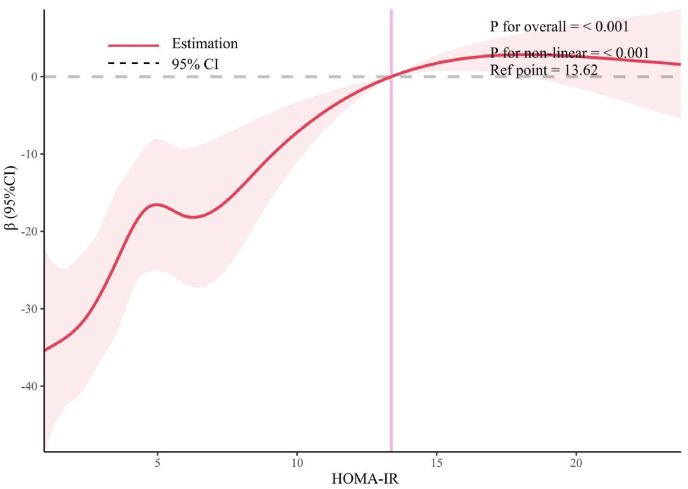
Examination of the nonlinear association between HOMA-IR and CAP. The RCS model adjusted for gender; age; race; education; ratio of family income to poverty; smoker; alcohol; hypertension; diabetes and physical activity.

Fig. 7 illustrates that after adjusting for variables such as gender, age, race, education, income, abdominal obesity, smoking, alcohol consumption, hypertension, diabetes, and physical activity, multivariate linear regression shows that CAP is significantly higher in Q3 and Q4 compared to Q1, with coefficients (β) of 17.619 (95 %CI = 8.974–26.265) for Q3 and 20.996 (95 %CI = 11.490–30.502) for Q4. Post-PSM, the difference in LSM between Q3, Q4, and Q1 increases considerably, with β values of 24.035 (95 %CI = 10.477–37.594) for Q3 and 32.704 (95 %CI = 19.459–45.950) for Q4. The mediation model was applied to the association between HOMA-IR and CAP (Fig. 8). The analysis revealed a significant total effect (β = 1.21, 95 % CI: 0.61 to 1.78, P < 0.001). Both the ADE (β = 0.97, 95 % CI: 0.39 to 1.53, P = 0.002) and the ACME (β = 0.24, 95 % CI: 0.08 to 0.43, P = 0.002) were statistically significant, accounting for a mediation proportion of 19.62 %. These results demonstrate that BMI also serves as a partial mediator in the relationship between HOMA-IR and CAP. RCS analysis, as depicted in Fig. 9, indicates a non-linear relationship between HOMA-IR and CAP (P for non-linear <0.001), identifying a threshold effect at a HOMA-IR value of 13.62. Below this threshold, the association remains significant (β = 2.624, 95 %CI = 1.370–3.878), while above it, the association diminishes and becomes non-significant (β = 0.527, 95 %CI = −1.168-2.223), as detailed in Table 4. These findings highlight that high HOMA-IR values do not correlate with increased CAP once a certain threshold is surpassed.

**Table 4:** Threshold effect analysis of HOMA-IR on the Controlled attenuation parameter using the two-piecewise linear regression model.

CAP	
Parameters	Adjusted β(95 %CI),P-value
**All participants**	
Fitting by the standard linear model	**1.575(0.952,2.198)<0.001**
**Fitting by the two-piecewise linear model**	
Inflection point	13.62
HOMA-IR≤13.62	**2.624(1.370,3.878)<0.001**
HOMA-IR>13.62	0.527(-1.168,2.223)0.529

Adjusted for:gender; age; race; education; ratio of family income to poverty; abdominal obesity; smoker; alcohol; hypertension; diabetes; physical activity.

## 4 Discussion

In this cross-sectional study analyzing the NHANES 2017–2020 dataset, we identified a robust association between the HOMA-IR and MASH prevalence in the MASLD population. This research uniquely identified a non-linear association between HOMA-IR and MASH prevalence, demonstrating an increase in MASH cases in parallel with rising HOMA-IR levels up to a certain threshold, beyond which the prevalence rate of MASH plateaus. Additionally, our findings show a positive correlation between HOMA-IR and both LSM and CAP. We found that the association curve is non-linear for HOMA-IR with LSM,CAP, with LSM,CAP levels not increasing after the threshold. Moreover, BMI mediated the associations of HOMA-IR with LSM and CAP.

MASH, part of the spectrum of MASLD, is driven by the accumulation of triglycerides in the liver, often coupled with other cardiometabolic disturbances [6,38]. Notably, our findings align with histology-based studies demonstrating HOMA-IR as an independent predictor of steatohepatitis and fibrosis [39]. The underlying mechanism, (IR, plays a crucial role not only in hepatic steatosis but also in the progression to more severe conditions like inflammation and fibrosis. During insulin resistance, the liver's ability to suppress gluconeogenesis is compromised, yet lipogenesis continues, leading to fat accumulation [9,40,41].This excess fat can impair mitochondrial function, increase ROS, and promote lipid peroxidation, further damaging liver cells and potentially exacerbating into chronic inflammation and liver damage [42,43]. Additionally, insulin resistance in adipose tissue enhances the release of free fatty acids (FFAs), particularly in fasting states, contributing to the disturbance of glucose homeostasis [44,45].Elevated FFAs lead to fat accumulation in the liver and other tissues like cardiac and skeletal muscles, exacerbating the metabolic dysfunction. The condition of hyperinsulinemia and hyperglycemia in NASH patients further intensifies hepatic lipogenesis, worsening the liver condition [[46], [47], [48]]. Moreover, MASLD can perpetuate a cycle of insulin resistance by fueling inflammation and lipotoxicity, thereby exacerbating the disease [49,50]. The systemic inflammatory milieu associated with IR is reflected in alterations of readily available biomarkers, as seen in the changes in complete blood count parameters in diabetic patients [16]. Furthermore, the association between elevated Galectin-3 levels and GDM [15] parallels the chronic low-grade inflammation that drives the progression from MASLD to MASH. The far-reaching consequences of metabolic dysregulation are also evident in its impact on endocrine axes beyond glucose metabolism, such as thyroid function and its subsequent effects on fertility [17,18]. This underscores that the metabolic dysfunction in MASLD is not isolated to the liver but is part of a systemic disorder. Research also indicates that organ-specific hormone imbalances, like elevated fetuin-A and decreased adiponectin levels, contribute distinctively to the pathophysiology of MASLD, linking specific organokine dysregulation with insulin resistance [[51], [52], [53], [54]].Addressing insulin sensitivity through interventions like ketogenic diets or metformin has shown promise in improving NAFLD and other metabolic dysfunctions, suggesting potential therapeutic avenues for managing and potentially reversing the impacts of insulin resistance in liver disease [[55], [56], [57], [58], [59]].

Our findings on the positive effect of insulin resistance on liver fibrosis are in consistent with previous studies. For example, the cross-sectional multicenter study including 361 participants showed that insulin resistance was an independent predictor of advanced liver fibrosis in nondiabetic patients with NAFLD [54].Furthermore, similar results were also observed in a cross-sectional study [60].The underlying mechanism may be due to the pro-inflammatory impact of insulin resistance [61].Insulin enhances the activity of natural killer cells, which inhibit the proliferation of hepatic stellate cells and attenuating liver fibrogenesis. Instead, insulin resistance stimulates the proliferation of hepatic stellate cells, elevates the production of connective tissue growth factor, and triggers the progression to liver fibrosis [62]. It also drives cirrhosis by promoting the synthesis of transforming growth factor beta 1, a pivotal agent in the development of liver fibrosis [63]. Elevated HOMA-IR levels may represent impaired NK cell function, thus contributing to liver fibrosis [64]. The underlying mechanisms of liver fibrosis in insulin resistance can be partially explained by obesity. Our mediation analysis confirms that BMI acts as a partial mediator, supporting insulin resistance promotes liver injury via increased adiposity. Excessive visceral fat is strongly associated with adipose insulin resistance (Adipo-IR). An increase in BMI and adipose tissue directly correlates with a higher incidence of diabetes, highlighting the role of abdominal obesity in exacerbating insulin resistance [65]. Obesity alters the profile of adipokines (hormones secreted by adipose tissue), such as adiponectin and leptin. Typically, adiponectin levels decrease in obesity, whereas leptin levels increase. Adiponectin has anti-inflammatory and insulin-sensitizing properties; its decrease may exacerbate insulin resistance and inflammation. Elevated leptin contributes to the activation of hepatic stellate cells and fibrogenesis, further linking obesity to liver fibrosis [66,67].

Our study boasts several strengths. Primarily, its utilization of the nationally representative NHANES database confers a significant advantage. Furthermore, the research conducts a comprehensive analysis of the association between HOMA-IR and not only MASH but also liver steatosis and fibrosis. Additionally, by adjusting for potential confounders and using PSM, we have enhanced the accuracy of our findings. We employed RCS to explore potential nonlinear relationships between HOMA-IR and MASH, LSM, CAP. Our analyses also investigated the mediating role of obesity on the relationship between insulin resistance and liver steatosis, fibrosis. Crucially, delineating the precise relationship between HOMA-IR and MASH enables the early identification of individuals at high prevalence for MASH. Active intervention of obesity may play an important role in reduce the severity of hepatic steatosis and fibrosis in adults with insulin resistance.

Our study faces several limitations. Firstly, the gold standard for assessing insulin resistance, the high insulin glucose clamp technique, is impractical for large-scale use due to its complexity and high costs. Instead, we used the HOMA-IR method, which is simpler and more feasible for broad applications but less accurate in certain populations such as those with advanced liver disease or low BMI, and it does not measure peripheral insulin resistance [68]. While we demonstrate HOMA-IR's association with MASH, we recognize that implementing insulin resistance assessment in routine MASLD care requires balancing added complexity against clinical utility. Future studies should evaluate simplified proxies (e.g., triglyceride-glucose index) that capture similar pathophysiology with greater practicality. To improve upon this, future research could integrate additional indices like QUICKI, the Matsuda Index from oral glucose tolerance tests, and the adipose tissue insulin resistance index for a more comprehensive assessment [[69], [70], [71]].Secondly, using the FAST score may introduce misclassification risk compared to biopsy, particularly false-positives and lower accuracy in obese individuals [72,73]. Despite this, FAST's integration of fibrosis, steatosis, and inflammation renders it superior to the fibrosis-focused FIB-4 for identifying high-risk MASH [74]. Therefore, while FIB-4 excels in initial triage [75], FAST provides superior secondary risk stratification [76]. Thirdly, while our study contributes broad insights into insulin resistance, it does not address the specific effects on different organs and their roles in diseases like hepatic steatosis and fibrosis. Future studies should explore organ-specific insulin resistance more thoroughly using techniques like [6,6-2] H2-glucose tracer and detailed oral glucose tolerance tests, though these methods are also costly and technically demanding [77,78]. Fourthly, our findings are based on the U.S. population and may not be directly applicable to other settings due to variations in diet, lifestyle, healthcare access, and genetics. Future research should aim to replicate these findings in diverse international contexts to enhance the global applicability of our insights into the relationships between insulin resistance, metabolic health, and liver disease. Fifthly, due to the inherent constraints of the cross-sectional design, our study cannot definitively establish temporal sequence or causality. While we hypothesize that insulin resistance contributes to the severity of MASH and liver fibrosis, the reverse causality remains a plausible alternative explanation. Longitudinal studies are necessary to better understand the progression of MASH and the causal impacts of insulin resistance. Incorporating a wider range of biomarkers and exploring diverse populations would deepen our understanding of disease mechanisms and help develop more effective strategies for managing insulin resistance and related metabolic disorders. Lastly, our mediation analysis, which posits BMI as a mediator in the pathway from HOMA-IR to liver injury, inherently assumes a specific directionality. However, the relationship between obesity and insulin resistance is bidirectional and often synergistic, creating a self-reinforcing cycle that complicates causal inference. To enhance the robustness of our findings, future studies should utilize longitudinal data to examine alternative causal sequences.

## 5 Conclusion

In summary, data from a nationally representative sample of American adults reveal a substantial correlation between HOMA-IR and MASH,LSM,CAP. As a obesity indicator, BMI mediated the relationships of HOMA-IR with LSM,CAP.This study provides evidence and operationalization for preventing MASH, liver steatosis and fibrosis through mitigating insulin resistance, as well as revealing obesity involved in the process. Further research is imperative to confirm these findings and to elucidate the mechanisms underlying such interactions, particularly in the MASLD population with concomitant insulin resistance.

## CRediT authorship contribution statement

**Xiao-Xuan Tang:** Writing – review & editing, Writing – original draft, Visualization, Validation, Supervision, Software, Resources, Methodology, Formal analysis, Data curation, Conceptualization. **Rui Wu:** Formal analysis, Data curation. **Jun-Hui Chen:** Methodology, Investigation, Formal analysis. **Feng-Lan Wang:** Visualization, Validation, Supervision, Data curation. **Sai-Li Zhao:** Validation, Investigation, Data curation. **Jie Lu:** Writing – review & editing, Writing – original draft, Visualization. **Jian Qin:** Writing – review & editing, Writing – original draft, Visualization, Methodology. **Duan-Ming Zhuang:** Writing – review & editing, Writing – original draft, Visualization, Validation, Supervision, Software, Resources, Project administration, Methodology. **Bin Zhang:** Writing – review & editing, Visualization, Validation, Resources.

## Ethics approval and consent to participate

This ethics review board of the National Center for Health Statistics approved all NHANES protocols.

## Consent for publication

Not applicable.

## Availability of data and materials

The survey data are publicly available on the internet for data users and researchers throughout the world (https://www.cdc.gov/nchs/nhanes/).

## Funding

Funded by the Nanjing 10.13039/100018696Health Science and 10.13039/100006180Technology Development Foundation (YKK24073), 10.13039/501100004608Natural Science Foundation of Jiangsu Province, China (BK20191119) and 10.13039/501100013059Jiangsu Provincial Medical Youth Talent (QNRC2016031).

## Competing interest

The authors declare that they have no competing interests.
