# Supplementary material for: Association between HOMA-IR and metabolic dysfunction-associated steatohepatitis in U.S. adults with MASLD
Source: Metabol Open. 2025 Sep 29;28:100402. doi: 10.1016/j.metop.2025.100402 (PMC12524561; doi:10.1016/j.metop.2025.100402)
Supplement: Multimedia component 1 [file mmc1.docx]

STROBE Statement—checklist of items that should be included in reports of observational studies

|  | Item No. | Recommendation | Page  No. | Relevant text from manuscript |
| --- | --- | --- | --- | --- |
| **Title and abstract** | 1 | (*a*) Indicate the study’s design with a commonly used term in the title or the abstract |  | This study introduces in the methodology section of the abstract that it is a cross-sectional study. In the abstract, we provide a detailed explanation of how this article investigates the relationship between insulin resistance and MASH using the NHANES database. |
|  |  | (*b*) Provide in the abstract an informative and balanced summary of what was done and what was found |  | The abstract of the article provides a detailed description of how the NHANES database was used to identify individuals with MASLD, and through logistic regression and restricted cubic spline analysis, it was found that insulin resistance increases the risk of developing MASH in MASLD patients.In addition, through mediation analysis, we found that insulin resistance can mediate hepatic steatosis and fibrosis through obesity. |
| Introduction | | | |  |
| Background/rationale | 2 | Explain the scientific background and rationale for the investigation being reported |  | Insulin resistance is not only associated with hepatic steatosis, but also with the progression of inflammation and liver fibrosis. To clarify whether insulin resistance increases the risk of MASH in the MASLD population, we used the NHANES database to investigate the relationship between HOMA-IR and MASH.To investigate the mediating role of BMI in the relationship between HOMA-IR and hepatic steatosis and fibrosis, we conducted a mediation analysis. |
| Objectives | 3 | State specific objectives, including any prespecified hypotheses |  | The main research objective of this article is to clarify whether insulin resistance increases the risk of MASH, liver steatosis, and fibrosis in the MASLD population.In addition, this article also investigated the mediating role of BMI in the association between HOMA-IR and LSM,CAP. |
| Methods | | | |  |
| Study design | 4 | Present key elements of study design early in the paper |  | The research population of this article comes from the NHANES database, and a cross-sectional study is conducted to explore the relationship between HOMA-IR and the risk of MASH. |
| Setting | 5 | Describe the setting, locations, and relevant dates, including periods of recruitment, exposure, follow-up, and data collection |  | The study population of this article is the American adult population with MASLD from 2017 to 2020. The HOMA-IR exposure factor in this article is calculated based on the patient's fasting blood glucose and fasting insulin. The data in this article comes from the NHANES database. |
| Participants | 6 | (*a*) *Cohort study*—Give the eligibility criteria, and the sources and methods of selection of participants. Describe methods of follow-up  *Case-control study*—Give the eligibility criteria, and the sources and methods of case ascertainment and control selection. Give the rationale for the choice of cases and controls  *Cross-sectional study*—Give the eligibility criteria, and the sources and methods of selection of participants |  | This article is a cross-sectional study that uses the NHANES database to determine MASLD and MASH. In the methodology section, we provided a detailed introduction to the inclusion and exclusion criteria of the study, such as excluding patients who drank excessively based on their alcohol consumption data. |
|  |  | (*b*) *Cohort study*—For matched studies, give matching criteria and number of exposed and unexposed  *Case-control study*—For matched studies, give matching criteria and the number of controls per case |  | This article is a cross-sectional study. |
| Variables | 7 | Clearly define all outcomes, exposures, predictors, potential confounders, and effect modifiers. Give diagnostic criteria, if applicable |  | In this article, the main research outcome is whether the patient has MASH, and the diagnosis of MASH requires the following conditions to be met simultaneously: (1) the patient needs to have hepatic steatosis; (2) Exclude other chronic liver diseases; (3) Meet at least one cardiaometabolic standard; (4) FibroScan Assessment of Steatohepatitis (FAST) score>0.35. The exposure of this article is HOMA-IR, which is (fast blood glucose [mmol/L] x fast insulin [μ U/mL])/22.5. The potential confounding factors adjusted by this paper are age, gender, race, education, income, BMI, smoking, drinking, hypertension, diabetes and exercise. The diagnosis of confounding factors was determined through previous literature and data from the NHANES database. |
| Data sources/ measurement | 8* | For each variable of interest, give sources of data and details of methods of assessment (measurement). Describe comparability of assessment methods if there is more than one group |  | *The data in this article comes from the NHANES database, and all research data in the article have been determined through previous literature and studies.* |
| Bias | 9 | Describe any efforts to address potential sources of bias |  | To control for bias, we used weighted multiple logistic regression to determine the relationship between HOMA-IR and MASH, with adjusted covariates including age, gender, race, and education level. To further improve the reliability of the study, we used restricted cubic splines and propensity score matching. |
| Study size | 10 | Explain how the study size was arrived at |  | Since the sample size was determined based on available data,  no prior statistical power calculation was conducted. |

Continued on next page

| Quantitative variables | 11 | Explain how quantitative variables were handled in the analyses. If applicable, describe which groupings were chosen and why |  | This article divides the quantitative data HOMA-IR into four groups. |
| --- | --- | --- | --- | --- |
| Statistical methods | 12 | (*a*) Describe all statistical methods, including those used to control for confounding |  | This paper uses weighted multivariate logistic regression to assess the relationship between HOMA-IR and MASH. The adjusted confounding factors include age, gender, race, education, income, BMI, smoking, drinking, hypertension, diabetes and exercise. In addition, we also used restricted cubic spline and propensity score matching to investigate the relationship between HOMA-IR and MASH.Finally, to investigate the mediating effect of BMI on the association between HOMA-IR, LSM, and CAP, we conducted casual mediation analysis. |
|  |  | (*b*) Describe any methods used to examine subgroups and interactions |  | This article did not conduct subgroup analysis. |
|  |  | (*c*) Explain how missing data were addressed |  | We have deleted the missing data. |
|  |  | (*d*) *Cohort study*—If applicable, explain how loss to follow-up was addressed  *Case-control study*—If applicable, explain how matching of cases and controls was addressed  *Cross-sectional study*—If applicable, describe analytical methods taking account of sampling strategy |  | Before propensity score matching, the sample data depends on the total number of available data. After matching the propensity score, we matched the patients according to their age, gender, and BMI, and 277 individuals were successfully matched between the non-MASH (n=181) and high risk MASH (n=96) groups. |
|  |  | (*e*) Describe any sensitivity analyses |  | We conducted sensitivity analysis using propensity score matching. |
| Results | | | | |
| Participants | 13* | (a) Report numbers of individuals at each stage of study—eg numbers potentially eligible, examined for eligibility, confirmed eligible, included in the study, completing follow-up, and analysed |  | We used a flowchart to report the number of individuals at each research stage. |
|  |  | (b) Give reasons for non-participation at each stage |  | In order to identify the population of MASLD and exclude those with missing data, we removed some populations. |
|  |  | (c) Consider use of a flow diagram |  | This article uses a flowchart in the methodology section to determine how the research population is determined. |
| Descriptive data | 14* | (a) Give characteristics of study participants (eg demographic, clinical, social) and information on exposures and potential confounders |  | In Table 1, we describe in detail the age, gender, race, BMI, income, LSM, smoking, drinking, hypertension, diabetes, exercise and HOMA-IR of patients in MASH and non MASH populations. |
|  |  | (b) Indicate number of participants with missing data for each variable of interest |  | We have removed the missing data, so there are no missing data in this article. |
|  |  | (c) *Cohort study*—Summarise follow-up time (eg, average and total amount) |  | This article is a cross-sectional study, not a cohort study. |
| Outcome data | 15* | *Cohort study*—Report numbers of outcome events or summary measures over time |  |  |
|  |  | *Case-control study—*Report numbers in each exposure category, or summary measures of exposure |  |  |
|  |  | *Cross-sectional study—*Report numbers of outcome events or summary measures |  | *This article is a cross-sectional study with 97 MASH patients and 603non MASH patients.* |
| Main results | 16 | (*a*) Give unadjusted estimates and, if applicable, confounder-adjusted estimates and their precision (eg, 95% confidence interval). Make clear which confounders were adjusted for and why they were included |  | The adjusted multivariate logistic regression shows a significant association between HOMA-IR and the overall prevalence of high-risk MASH, as determined using FAST scores of ≥0.35 and ≥0.67. For Q3 and Q4, the odds ratios (ORs) are significantly higher compared to Q1. Specifically, for a FAST score of ≥0.35, the OR is 3.784 (95%CI = 1.406-10.182) for Q3 and 14.715 (95%CI = 1.120-193.265) for Q4. For a FAST score of ≥0.67, the OR increases to 5.942 (95%CI = 2.117-16.679) for Q3 and 17.621 (95%CI = 1.714-181.135) for Q4.PSM analysis further supports these findings, demonstrating even higher ORs in Q3 and Q4 relative to Q1. For a FAST score of ≥0.35, the OR post-PSM is 5.028 (95%CI = 1.629-15.520) for Q3 and 22.042 (95%CI = 1.605-302.751) for Q4; for a FAST score of ≥0.67, the OR post-PSM is 9.118 (95%CI = 2.825-29.426) for Q3 and 36.508 (95%CI = 1.383-963.486) for Q4 |
|  |  | (*b*) Report category boundaries when continuous variables were categorized |  | We divided study participants into quartiles based on their HOMA-IR values, with Quartile 1 (Q1) ranging from 0.50 to 2.85, Quartile 2 (Q2) from 2.86 to 4.63, Quartile 3 (Q3) from 4.64 to 7.40, and Quartile 4 (Q4) from 7.41 to 65.20. |
|  |  | (*c*) If relevant, consider translating estimates of relative risk into absolute risk for a meaningful time period |  | This article is a cross-sectional study and cannot convert relative risk into absolute risk. |

Continued on next page

| Other analyses | 17 | Report other analyses done—eg analyses of subgroups and interactions, and sensitivity analyses |  | This article did not conduct subgroup analysis. In addition, we also conducted propensity score matching according to the patient's age, gender, BMI and diabetes. As a sensitivity analysis, propensity score matching is used to evaluate the robustness of the study. |
| --- | --- | --- | --- | --- |
| Discussion | | | | |
| Key results | 18 | Summarise key results with reference to study objectives |  | In summary, data from a nationally representative sample of American adults reveal monitoring HOMA-IR can facilitate early MASH detection and intervention, potentially curbing disease progression. Additionally, targeting both insulin resistance and BMI could effectively mitigate MASH incidence and severity, emphasizing the importance of integrated therapeutic strategies. |
| Limitations | 19 | Discuss limitations of the study, taking into account sources of potential bias or imprecision. Discuss both direction and magnitude of any potential bias |  |  |
| Interpretation | 20 | Give a cautious overall interpretation of results considering objectives, limitations, multiplicity of analyses, results from similar studies, and other relevant evidence |  | Our study is subject to certain limitations. Primarily, the diagnosis of MASLD is based on CAP assessments rather than liver biopsies. Yet, the breadth of our sample size helps to mitigate this constraint. Secondly, the cross-sectional nature of our investigation precludes us from inferring causality. Additionally, despite accounting for numerous covariates to address the multifactorial aspects influencing MASH, residual confounding may persist post-adjustment. Lastly, although our findings suggest a nonlinear association between HOMA-IR and MASH, the precise underlying mechanisms remain unexplored, which will guide our future research work. |
| Generalisability | 21 | Discuss the generalisability (external validity) of the study results |  | The data in this article comes from the NHANES database. After weighting the data, this study can be extended to all American adults with MASLD. |
| Other information | |  | | |
| Funding | 22 | Give the source of funding and the role of the funders for the present study and, if applicable, for the original study on which the present article is based |  | This article was funded by the Nanjing Health Science and Technology Development Foundation (YKK24073), Natural Science Foundation of Jiangsu Province, China (BK20191119) and Jiangsu Provincial Medical Youth Talent (QNRC2016031). |

*Give information separately for cases and controls in case-control studies and, if applicable, for exposed and unexposed groups in cohort and cross-sectional studies.

**Note:** An Explanation and Elaboration article discusses each checklist item and gives methodological background and published examples of transparent reporting. The STROBE checklist is best used in conjunction with this article (freely available on the Web sites of PLoS Medicine at http://www.plosmedicine.org/, Annals of Internal Medicine at http://www.annals.org/, and Epidemiology at http://www.epidem.com/). Information on the STROBE Initiative is available at www.strobe-statement.org.
