# Supplementary material for: Association between HOMA-IR and metabolic dysfunction-associated steatohepatitis in U.S. adults with MASLD
Source: Metabol Open. 2025 Sep 29;28:100402. doi: 10.1016/j.metop.2025.100402 (PMC12524561; doi:10.1016/j.metop.2025.100402)
Supplement: Multimedia component 2 [file mmc2.docx]

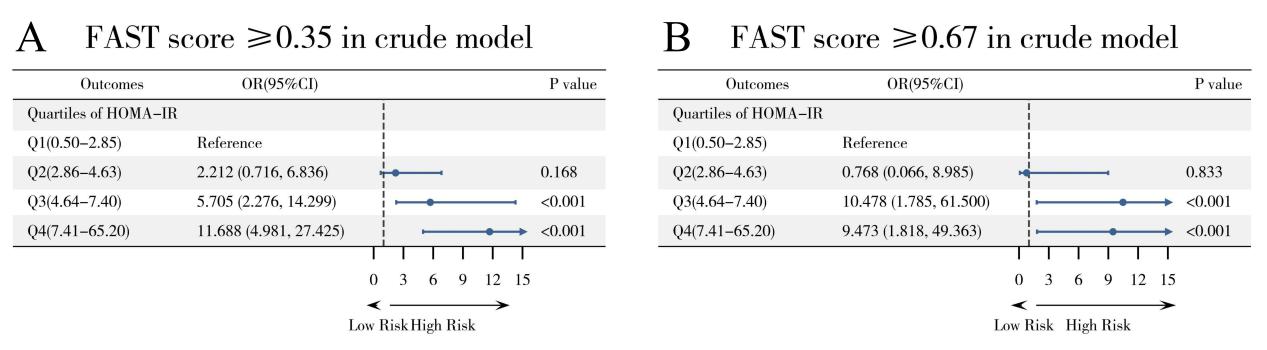


**Supplementary Figure 1**: Association between HOMA-IR and the prevalence of MASH (Panel A and B: using FAST score cutoffs of ≥0.35 and ≥0.67) in crude model. The crude model was adjusted for no covariates.
